# Indication Alerts Intercept Drug Name Confusion Errors during Computerized Entry of Medication Orders

**DOI:** 10.1371/journal.pone.0101977

**Published:** 2014-07-15

**Authors:** William L. Galanter, Michelle L. Bryson, Suzanne Falck, Rachel Rosenfield, Marci Laragh, Neeha Shrestha, Gordon D. Schiff, Bruce L. Lambert

**Affiliations:** 1 Department of Medicine, University of Illinois at Chicago (UIC), Chicago, Illinois, United States of America; 2 Department of Pharmacy Practice UIC, Chicago, Illinois, United States of America; 3 College of Medicine, University of Illinois, Chicago, Illinois, United States of America; 4 Department of Pharmacy Administration UIC, Chicago, Illinois, United States of America; 5 Department of Medicine, Harvard University, Boston, Massachusetts, United States of America; 6 Department of Communication Studies and Center for Communication and Health, Northwestern University, Chicago, Illinois, United States of America; Universidad de Valladolid, Spain

## Abstract

**Background:**

Confusion between similar drug names is a common cause of potentially harmful medication errors. Interventions to prevent these errors at the point of prescribing have had limited success. The purpose of this study is to measure whether indication alerts at the time of computerized physician order entry (CPOE) can intercept drug name confusion errors.

**Methods and Findings:**

A retrospective observational study of alerts provided to prescribers in a public, tertiary hospital and ambulatory practice with medication orders placed using CPOE. Consecutive patients seen from April 2006 through February 2012 were eligible if a clinician received an indication alert during ordering. A total of 54,499 unique patients were included. The computerized decision support system prompted prescribers to enter indications when certain medications were ordered without a coded indication in the electronic problem list. Alerts required prescribers either to ignore them by clicking OK, to place a problem in the problem list, or to cancel the order. Main outcome was the proportion of indication alerts resulting in the interception of drug name confusion errors. Error interception was determined using an algorithm to identify instances in which an alert triggered, the initial medication order was not completed, and the same prescriber ordered a similar-sounding medication on the same patient within 5 minutes. Similarity was defined using standard text similarity measures. Two clinicians performed chart review of all cases to determine whether the first, non-completed medication order had a documented or non-documented, plausible indication for use. If either reviewer found a plausible indication, the case was not considered an error. We analyzed 127,458 alerts and identified 176 intercepted drug name confusion errors, an interception rate of 0.14±.01%.

**Conclusions:**

Indication alerts intercepted 1.4 drug name confusion errors per 1000 alerts. Institutions with CPOE should consider using indication prompts to intercept drug name confusion errors.

## Introduction

Confusions between drug names that look and sound alike are a common, costly and persistent type of medication error (e.g., hydroxyzine/hydralazine, Fosamax/Flomax, Durasal/Durezol) [1]–[3]. Wrong drug errors, of which drug name confusions are thought to be the most common type, occur at the rate of at least one per thousand prescriptions in the inpatient [4]–[6] and outpatient settings [7]. Regulatory agencies and the pharmaceutical industry have taken steps to reduce the risk of these errors, [8], [9] but few interventions have produced convincing evidence of sustained improvement in wrong drug error rates, especially at the point of prescribing. Barcoding has been shown to be useful in preventing wrong drug errors, but at the time of administration [6]. So-called ‘tall-man’ lettering, where capitalization is used to make similar names more distinctive (e.g., hydrOXYzine/hydrALAzine), has had mixed success in lab experiments, but there is no published evidence of its real-world impact [10]–[12]. Developing additional effective methods to reduce the rate of drug name confusion errors is an important medication safety priority.

As part of a separate project, we developed and implemented a set of clinical decision support (CDS) alerts to prompt prescribers to add problems to the electronic medical record (EMR) problem list when the prescriber ordered selected medications in the absence of a documented indication (e.g., prescribing metformin when diabetes was not on the problem list). We have previously shown that these indication alerts improve problem list documentation, with an acceptable error rate. The performance of such alerts varies across medications, and appears to be a function of the number of legitimate indications that exist for a given medication (i.e., those with many indications perform poorly; those with few indications perform well) [13]–[15].

We recently reported on the ability of indication alerts to intercept wrong patient errors at a rate of roughly 1 per 4000 alerted orders [16]. The mechanism by which the alerts intercept errors is not completely clear, but we typically find that the appearance of an alert forces the prescriber to take a brief “time out” in the medication order process. During this time, the prescriber may reflect on the patient, medication and indication in relation to one another, and this additional reflection potentially allows errors to be self-identified and self-corrected. Indication alerts may function in a similar way to intercept drug name confusion errors. If a clinician were to inadvertently select an incorrect medication due to a pick-list or memory error, she would have an opportunity to recognize the mistake when forced by the alert to interrupt the ordering process and review the selected drug's indication in the context of the current patient and the indication being suggested by the alert. The aim of this study was to quantify the extent to which indication-based alerts during computerized physician order entry (CPOE) of medications could help intercept imminent drug name confusion errors prior to completion of the order.

## Methods

### Setting

The University of Illinois Hospital and Health Sciences System (UI-Health) is comprised of a 450-bed tertiary care hospital, large multi-specialty ambulatory care clinic, emergency department and 19 community clinics. All clinical areas utilize a commercial EMR (*Millennium*, Cerner Corporation, Kansas City, MO) for problem lists, clinical notes, test results, medication lists and orders. The EMR allowed any clinician to update patient records and problem lists either as free text or using common, discrete-coded nomenclatures (*ICD-9 CM* or *SNOMED*). Almost all medication orders are placed by CPOE, which is associated with a commercially available CDS system (*Discern Expert*, Cerner Corporation), described previously for indication alerts [12] and other types of alerts [13], [17], [18].

### Clinical Decision Support

**Figure 1:**
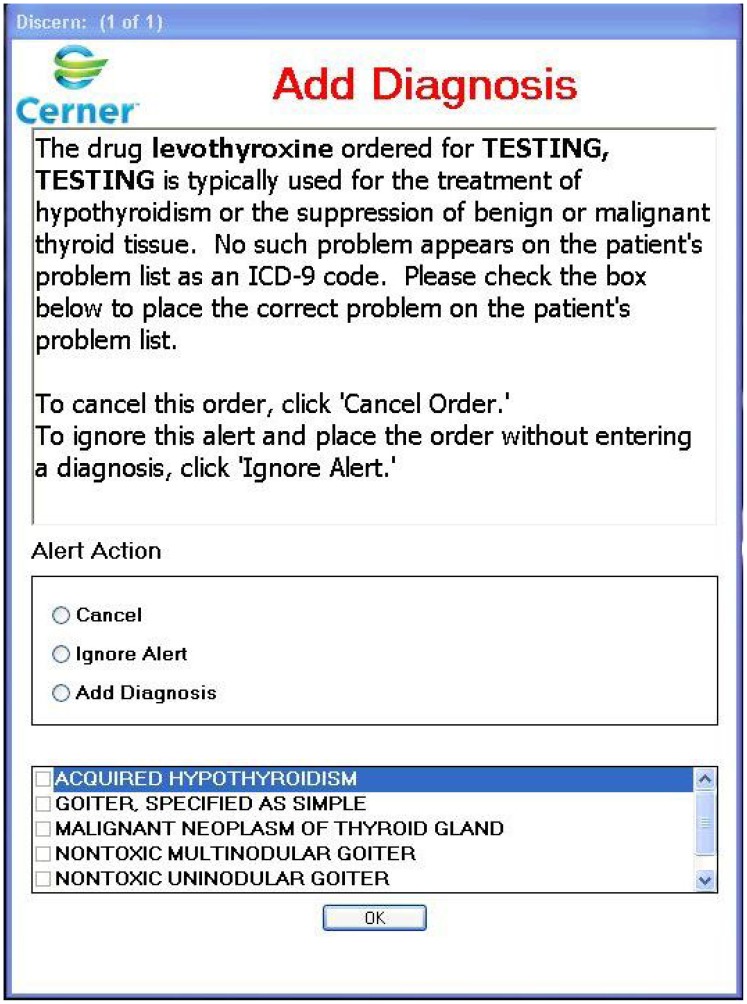
Example Indication Alert for Levothyroxine.

In the CDS system we developed and implemented, orders for a selected subset of medications (Table 1) triggered an alert for the clinician to update the medical record if the patient's electronic problem list did not contain an active condition indicated by that medication (e.g., prescribing metformin when diabetes is not on the problem list) [13]. Depending on the medication, alerts displayed one or more possible diagnoses (Figure 1). The clinician could select one or more of the offered indications, ignore the alert, or cancel the order. Once selected, indications were added automatically to the patient's problem list in the EMR. The medications were selected to maximize accurate problem list placement, due to their frequent use and relatively limited indications.

**Table 1:** Medications used as Indication Alert Triggers.

Albuterol, aliskiren, alpha blockers, amiloride, angiotensin converting enzyme inhibiters, angiotensin receptor blockers, aspirin-dipyridamole, beta blockers, bisphosphonates, calcium channel blockers, clonidine, cholestyramine, coagulation factor VIIa, ezetimibe, fibric acids, intravenous immune globulin, fluticasone, fluticasone/salmeterol, furosemide, glimepiride, guanfacine, HMG-CoA reductase inhibitors, hydralazine, insulins, isosorbide dinitrate, levothyroxine, liotrix, metformin, methyldopa, metolazone, minoxidil, nateglinide, niacin, nitroprusside, non-nucleoside reverse transcriptase inhibitors, nucleoside reverse transcriptase inhibitors, nucleotide analog reverse-transcriptase inhibitors, nucleotide reverse transcriptase inhibitors, pioglitazone, protease inhibitors, proton pump inhibitors, red yeast rice, repaglinide, rosiglitazone, selective serotonin reuptake inhibitors, spironolactone, sulfonylureas, thiazides, thyroid desiccated, tiotropium, triamterene

Specific sets of alerts were designed for selected diagnostic categories and implemented from April 2006 through July 2010. Almost all alerts were active throughout the medical center: inpatient, outpatient and the emergency department (ED). Certain medications were only active in the outpatient setting since their use in the inpatient setting was non-specific for a particular disease (e.g., insulins).

### Detecting Intercepted Errors

In order to identify potential drug name confusion errors, we analyzed all indication alerts triggered from April 2006 through February 2012 to identify instances of orders that may have been cancelled due to physician recognition of the error. For an alert to be confirmed by chart review as an actual error, all of the following criteria had to be met, with steps (a), (b), and (c) done by computer program

- An order was started but not completed for a given medication.
- The same prescriber completed an order for another medication on the same patient within 5 minutes of the initial order.
- The canceled medication name and subsequent completed medication order name had a Bisim [19] and Editex [20] similarity score greater than or equal to 0.40. The value 0.40 was chosen somewhat arbitrarily, based on examination of the distribution of similarity scores for a large set of name pairs known to be confusing. Bisim and Editex are orthographic, “look alike” and phonetic, “sound alike”, measures [18]–[19].
- Two experienced clinicians, blinded to each other's review, determined that the medication whose order was cancelled did not have a plausible indication for use, either through documentation in the problem list or by review of clinical documentation.

If either reviewer determined that the chart documented a legitimate indication for the initial medication order, the case was not counted as an intercepted error. This approach is more conservative than using a third reviewer, since any disagreement was sufficient to disqualify a case as an error. The presence of a chronic indication was not always a reason to rule a case out as an intercepted error. This exception was necessary because some diseases, such as hypertension, are so highly prevalent. Truly erroneous attempts at ordering antihypertensives would frequently have to be ruled out as intercepted errors because of the ubiquity of hypertension diagnoses. To make an assessment in cases where the patient had a prior, documented indication, reviewers judged whether the use of the medication was plausible *at the time of the order*. For instance, a patient with a history of hypertension who is admitted for sepsis and hypotension would not be given an order for a new antihypertensive despite the prior history. A reviewer could deem this as a non-plausible indication at the time of the order.

We also excluded cases if the reviewers determined the medication order was part of medication reconciliation. This was necessary since the medication reconciliation lists in the EMR listed medications alphabetically, so the fact that metronidazole was selected after metformin was cancelled could simply be because it was the next medication to reconcile, and it might not represent any drug name confusion.

In the inpatient setting, medications can only be ordered in our EMR by their generic name. In outpatient and ED areas, medications could be ordered by either brand or generic names. For the retrospectively reviewed, canceled orders, we could not retrieve whether the medication was ordered generic or branded. In order to better determine the likely name of the ordered medications in the ambulatory settings, we reviewed prescription data at the time of the suspected error to look into the relative use of generic versus brand names. If prescriptions were written 90% or more of the time as either generic or branded, then we evaluated that medication solely by the medication name that was most commonly used. As it happened, all of the names examined during chart review were prescribed consistently as either branded or generic greater than 90% of the time.

### Statistical Analysis Plan

We computed the rate of intercepted errors as the number of intercepted errors divided by the total number of alerts. We used multivariable logistic regression to examine the effect of potential covariates on the dependent variable, the probability of an intercepted error. We considered prescriber type (resident, attending, other), patient location (inpatient, emergency department, outpatient, other), and work shift (7am-5pm, 5pm-midnight, midnight-7am). Because errors with fluticasone/fluticasone nasal dominated the results, the analysis was done with both the full cohort and then again excluding fluticasone/fluticasone nasal cases. The threshold for statistical significance was alpha = 0.05. This study was approved by the Institutional Review Board at the University of Illinois Hospital and Health Sciences System (UI-Health). We obtained a waiver of informed consent for both the patient and clinician participants.

## Results

We analyzed a total of 127,458 indication alerts. The inpatient setting accounted for 46% of the orders, followed by 35% in the ambulatory setting, 16% from the ED and 3% other. Resident physicians accounted for 72% of the orders, attending physicians 21%, nurses 6%, and 1% other. In 2,410 (1.9%) instances the order was never completed (i.e., “abandoned”). Of these 2,410 abandoned orders, 731 met criteria (a), (b) and (c) in our definition of an intercepted error and were reviewed by clinicians. Chart review by two independent clinicians identified 176 instances in which the alert led to the order of a different, similar-sounding drug, by the same clinician, in the same patient, within five minutes, with no plausible indication for the initially ordered drug. This represented an error interception rate of 0.14±0.01% (176 intercepted errors out of 127,458 alerts), or 1.4 intercepted errors per 1000 alerted orders. The 176 errors were comprised of 39 unique pairs of drug names (Table 2). Ranked by overall number of errors, the most commonly intercepted pairs were fluticasone/fluticasone nasal, 100 (57%), metoprolol/metoclopramide, 16 (9%), hydralazine/diphenhydramine, 6 (3%), and nitroprusside/nitroglycerin, 6 (3%).

**Table 2:** Distribution of Drug Pairs in Intercepted Errors.

Canceled Order	Completed Order	No. Errors	No. Alerts	Error Rate (%)^a^	Std. Err. (%)
fluticasone	fluticasone-nasal	100	4565	2.19	0.22
metoprolol	metoclopramide	16	9936	0.16	0.04
nitroprusside	nitroglycerin	6	19	31.6	10.7
hydralazine	diphenhydramine	6	4346	0.14	0.06
propranolol	propofol	5	2931	0.17	0.08
nimodipine	famotidine	3	511	0.59	0.34
clonidine	famotidine	3	2350	0.13	0.07
metformin	metronidazole	3	6148	0.05	0.03
fluticasone-salmeterol	fluticasone-nasal	2	3166	0.06	0.04
hydralazine	hydromorphone	2	4346	0.05	0.03
hydralazine	hydroxyzine	2	4346	0.05	0.03
methyclothiazide	methylergonovine	1	1	100	0.00
minoxidil	minoxidil-topical	1	48	2.08	2.06
tenofovir	atenolol	1	101	0.99	0.99
metolazone	metoclopramide	1	132	0.76	0.75
ibandronate	ibuprofen	1	133	0.75	0.75
methyldopa	methylprednisolone	1	138	0.73	0.72
Actonel^b^	Actos	1	150	0.67	0.66
felodipine	Feldene	1	177	0.57	0.56
nimodipine	morphine	1	511	0.20	0.20
paroxetine	pyridoxine	1	1071	0.09	0.09
tiotropium	tenofovir	1	1581	0.06	0.06
nifedipine	prednisone	1	2111	0.05	0.05
clonidine	ranitidine	1	2350	0.04	0.04
lansoprazole	alprazolam	1	2381	0.04	0.04
propranolol	lansoprazole	1	2931	0.03	0.03
amlodipine	amitriptyline	1	3183	0.03	0.03
amlodipine	famotidine	1	3183	0.03	0.03
atorvastatin	multivitamin	1	3204	0.03	0.03
hydralazine	chlorzoxazone	1	4346	0.02	0.02
sertraline	tetracycline	1	4529	0.02	0.02
levothyroxine	levofloxacin	1	5642	0.02	0.02
hydrochlorothiazide	hydrocortisone	1	6040	0.02	0.02
hydrochlorothiazide	hydroxyzine	1	6040	0.02	0.02
metformin	metoprolol	1	6148	0.02	0.02
metformin	multivitamin	1	6148	0.02	0.02
metoprolol	ketorolac	1	9936	0.01%	0.01%
metoprolol	metronidazole	1	9936	0.01%	0.01%
simvastatin	simethicone	1	13625	0.01%	0.01%

a The interception rate is the number of errors (confirmed by clinician chart review) divided by the total number of alerts for that drug.

b In this pair, at the time of the alert, the branded names were most common, >90%.

Of the 39 unique pairs, the similarity between the confused meds was most commonly having the first 3 letters being identical, 20/39 (51%). Having neither the first or last 3 letters in common occurred in 10/39 (26%), while having the last 3 letters in common occurred in 9/39 (23%) of the pairs.

The rate of error interception varied by medication. For many drugs, like simvastatin, the rate was so low that it could not be measured with precision different than zero. Nitroprusside, not a commonly used medication, had a very high interception rate of roughly one intercepted error per 3 alerted orders. Two commonly used drugs with relatively high intercepted error rates were hydralazine (0.25±0.07%) and metoprolol (0.18±0.04%).

The distribution of intercepted errors as a function of initially ordered medication shown in Table 2 is dominated by fluticasone. These intercepted errors were an attempt to prescribe fluticasone nasal, which is most frequently an outpatient medication. To understand the relationship between the covariates and the outcome with and without the influence of the fluticasone cases, logistic regression was repeated, once including fluticasone cases and once without. The results are shown in Table 3. Comparison of the results with and without fluticasone demonstrates the effect of this one medication on the results.

**Table 3:** Association between Clinician Type, Location, Shift and Probability of an Intercepted Drug Name Confusion Error.

Variable^a^	With Fluticasone		Without Fluticasone	
	Odds Ratio	Confidence Interval	Odds Ratio	Confidence Interval
Clinician Type				
Resident	-	-	-	-
Attending	0.35^b^	0.23–0.54	0.69	0.30–1.56
Nurse	0.53^c^	0.28–0.996	0.69	0.21–2.26
Location				
Inpatient	-	-	-	-
Ambulatory	3.54^b^	2.51–5.00	0.51^c^	0.27–0.95
ED	0.27^c^	0.11–0.68	0.29^c^	0.11–0.72
OR	0.27	0.04–1.98	0.29	0.04–2.09
Shift				
Day	-	-	-	-
Evening	0.80	0.55–1.16	0.47^c^	0.23–.95
Overnight	0.74	0.44–1.27	1.05	0.52–2.12

a Resident physician, inpatient location and day shift were used as reference categories. Testing global null hypothesis for model with fluticasone, −2 log likelihood  = 2563.8, chi-square  = 94.9, p<.0001. For model without fluticasone, −2 log likelihood  = 1245.9, chi-square  = 24.6, p<.0001.

b p<0.001.

c p<0.05.

In both analyses, the likelihood of an intercepted error was numerically highest with resident physicians. This was a statistically reliable result in the complete data set and a non-significant trend when fluticasone was excluded. The ED had a significantly lower rate of intercepted errors both with and without fluticasone included in the analysis. The time of day as defined by the shift was only significantly related to the error interception rate when fluticasone was excluded, with the evening shift being associated with roughly half the rate of the day shift.

## Discussion

Drug name confusions are a relatively common and persistent source of medication errors. Even when the confusing pairs of names are well known, as they often are (e.g., hydroxyzine/hydralazine), errors have stubbornly resisted eradication. Safety experts suggest a variety of techniques for minimizing the risk: storing products with similar names in different locations, eliminating one of the two confusing products from the formulary, adding labels to shelves where products are stored, adding warnings to computer order entry systems, using mixed-case (“tall man”) lettering, altering labels and packages among other interventions^8^ In spite of the widespread use of some of these risk-reduction strategies, especially mixed-case lettering, only barcoding is strongly evidence-based [6]. One strategy that has not been widely discussed previously is to incorporate information from a patient's problem list and known drug indications into real-time decision support at the time orders are entered. We demonstrated here that indication alerts can intercept drug name confusion errors at a rate of just over one per thousand alerted orders. This is roughly the same rate at which wrong drug errors, of which name confusion errors are a subset, have been observed in inpatient and outpatient studies, though we hasten to add that the rate we report is only the rate of intercepted errors for a subset of drugs that had indication alerts turned on [4], [7].

Of the 39 unique pairs of names we identified in intercepted errors, all but 3 (Actonel/Actos, metformin/metronidazole, hydrozyzine/hydralazine) do not appear on the most widely circulated list of confusing drug names, suggesting that many more pairs of names are confused in practice than have been reported in published sources [21]. About half of the pairs had at least 3-letter similarity at the beginning of the names, while the others were split between 3 letter similarities as the end of the names and no 3 letter similarity at the beginning or end. The half of the pairs that had similarity at the beginning of the words accounted for 81% of the drug name confusions. Several error mechanisms may have been in play, including pick list errors, auto-fill errors and adjacency errors found either on main selection screens, drop down menus, order sets, or favorites lists.

We are in the process of analyzing our own order entry screens, using the identified errors as queries, to see if we can further isolate system vulnerabilities that increase risk of confusion. For the errors with dissimilar beginning of names, the error mechanisms are less clear, but are also likely to involve some combination of visual perception, auditory perception, short-term memory and motor control errors [22]. The fact that the error interception rate varied among pairs in intriguing and further analysis need to occur to understand the ramifications for order entry design.

The interception rate is a measure of the potential benefit of the intervention; however, changes in the interception rate do not necessarily reflect a change in the underlying error rate because of the unknown rate of *non-intercepted* errors. Prescribers must pay attention at the time of the alert if they are to recognize and correct errors. A clinician who is hurried and distracted when ordering may be more likely to produce a drug name confusion error, but may also be less likely to recognize the error even after the alert draws attention to the medication name, indication and patient name. If prescribers do not attend to alerts, the interception rate may be low despite a high rate of actual errors because many errors are not being intercepted. As such, the odds ratios reported in Table 3 are not meant to imply anything more than the performance of the intervention in intercepting errors. The data suggest that the intervention was able to intercept more errors for resident physicians as well as in the inpatient or ambulatory setting as compared to the ED. It does not mean that the *actual error rate* differed across these settings. The actual error rate remains unknown.

In addition to the interception rate, the value of the alerts should also be assessed by considering the severity of the potential harm prevented by intercepting certain errors. Although we did not conduct a formal assessment of the clinical severity of the intercepted errors, several, by their very nature, have the potential for significant harm. Examples include high-alert medications like metoprolol, propofol, morphine and others.

The results of this study, together with prior work by our group, suggest that indication alerts can have 3 beneficial effects: improvement of the problem list, [13] interception of wrong patient errors,^16^ and interception of drug name confusion errors. None of these benefits was found in a system operating across all medications, and, in the case of problem list additions, indication alerts for some medications are known to perform poorly [15]. In a recent analysis of all alerts for antihypertensive medications, the accuracy and yield of problem list additions varied greatly and was, at least in part, related to the number of potential indications associated with the medication being ordered [14].

To the prescribing clinician, the alert's function is to help with problem list placement. Error interception is rare and does not add any nuisance to the alerts. Documented variability in the effectiveness of indication-based alerts demonstrates that they must be designed thoughtfully. To minimize nuisance, great care must be given to selecting medications with a limited number of well-documented indications and to incorporating an exhaustive list of exclusion diagnoses so that alerts will not trigger when the patient has a legitimate indication.

## Limitations

We conducted the study in a hospital that has been using CPOE for more than 20 years and in a teaching environment where prescribers are accustomed to alerts. Many settings lack CPOE, EMR and electronic CDS, and virtually none prompt physicians with alerts about missing problems that are linked to drug orders, although the capability to perform CPOE with this type of decision support is becoming more widespread. As a result, the performance we observed may not be generalizable to settings unlike the one we studied. We used established measures (e.g., Editex and BiSim) to measure similarity between the initial name and the subsequently ordered name, but the threshold value we used to define what counted as a name confusion (as opposed to any other type of wrong drug error) was chosen empirically, based on an examination of the distribution of similarity scores for a large set of name pairs. Using a different threshold would impact the number of order changes we defined as drug name confusions. We did not interview the prescribers who intercepted their own errors, nor did we directly observe them during order entry, so our understanding of the precise mechanism by which the alerts functioned is incomplete. Finally, this analysis likely underestimated the true frequency of wrong drug orders since we suspect many alerts were ignored or suppressed by the computer when a plausible problem was on the problem list.

## Conclusions

Indication alerts have been shown to improve problem list documentation and to intercept wrong patient medication errors. In this study we demonstrated that indication alerts intercepted 1.4 drug name confusion errors per 1000 alerted orders. Institutions with CPOE should consider implementing indication prompts both to improve the quality of problem lists and to prevent drug name confusion errors and wrong patient errors. Further enhancements to maximize the benefit of this novel form of CDS while minimizing nuisance, likely through careful design and selection of medications, indications, prescribers, and locations, is necessary.
